# Effects of Continuous Use of Entonox in Comparison with Intermittent Method on Obstetric Outcomes: A Randomized Clinical Trial

**DOI:** 10.1155/2014/245907

**Published:** 2014-11-30

**Authors:** Jila Agah, Roya Baghani, Seid Hossein Safiabadi Tali, Yaser Tabarraei

**Affiliations:** ^1^Department of Obstetrics & Gynecology, Faculty of Medicine, Sabzevar University of Medical Sciences, Sabzevar, Iran; ^2^Department of Midwifery, Sabzevar University of Medical Sciences, Sabzevar, Iran; ^3^Department of Internal Medicine, Ghazvin University of Medical Sciences, Ghazvin, Iran; ^4^Department of Biostatistics and Health, Sabzevar University of Medical Sciences, Sabzevar, Iran

## Abstract

*Background*. Entonox (N_2_O_2_) which is an inhalational gas for relieving labor pain is commonly used intermittently; however some women are interested in continuous breathing in face mask. So we decided to compare the complications induced by two methods to find out whether it is safe to permit the mothers to use Entonox continuously or not. *Patients and Methods*. This randomized clinical trial was performed in Mobini Hospital, Sabzevar, Iran. 50 parturients used Entonox intermittently and 50 cases used it continuously during labor. Then obstetrical outcomes were analyzed in two groups by spss 17 software, *t*-test, and Chi^2^ while *P* < 0.05 was considered significant. *Results*. This study showed the mean duration of second stage of labor had no significant difference (*P* = 0.3). Perineal laceration was less in continuous group significantly (*P* = 0.04). Assisted vaginal birth was not different significantly (*P* = 0.4). Uterine atony had no significant difference in two groups (*P* = 0.2). Maternal collaboration in pushing and satisfaction were higher in continuous group significantly (*P* = 0.03), (*P* < 0.0001). Apgar score of neonates at first and fifth minute was acceptable and not different significantly in two groups (*P* = 0.3). *Conclusions*. Our study demonstrated continuous method is also safe. So, it seems reasonable to set mothers free to choose the desired method of Entonox usage.

## 1. Introduction

Over many centuries human has always persued finding a harmless way to relieve labor pain. Many methods have been used for this purpose; some of them constitute nonpharmaceutical methods such as relaxation therapy and others are applied by pharmaceutical agents [1]. Severity of labor pain depends on some factors such as psychological background of mother, size and presentation of fetus, whether labor is spontaneous or augmented, and rate of cervical dilatation in first stage [2]. Indeed, severe pain associated with maternal anxiety can lead to maternal exhaustion, frustration, and inability to push in terminal stages of labor. This process in addition to fetal damage can lead to maternal complications such as perineal lacerations, uterine atony, uterine rupture, and even maternal death [1–3].

Numerous methods are administered for decreasing labor pain. Gas inhalation is an old method which was firstly applied in the eighteenth century. By gradual development, Minitt invented a self-administrable machine for relieving pain in 1934. Entonox which is a composition of 50% N_2_O and 50% O_2_ compacted in cylindric containers was commercially introduced in 1961. During last decades, Entonox has gained considerable acceptance in many European and Asian countries [4, 5]. The mechanism of its action is probably release of endorphin and dopamine in the brain which modulates pain stimuli via descending spinal and nerve pathways and in consequence reduces labor pain to a tolerable level [2, 3, 5].

Entonox has many advantages; it is tasteless, odorless, colorless, easy to use, and with rapid onset and offset [3, 5, 6]. Its filtration is totally by lungs, which makes it harmless in the cases of hepatic and renal diseases as well [6]. Also it is cost effective and noninvasive with minimal side effects like drowsiness and vomiting which disappear a few minutes after discontinuation of gas [5]. It is worth mentioning that Entonox does not affect labor length and maternal pushing force during delivery [2, 3, 6].

Entonox which is administered by a self-use mask can be used intermittently or continuously. In the intermittent method the parturient breathes in the mask during contractions and puts it aside between them while in the continuous method she uses the mask permanently [7]. For several decades, almost all maternity centers use intermittent method due to fear of maternal complications by continuous method [8, 9]. However, the intermittent method has some disadvantages. The peak of analgesic effect of nitrous oxide lags the start of its administration by 50 seconds whereas the pain peak is 30 seconds from commencement of uterine contraction. So, for maximum perception of painless effect, the mother should use the mask at least 30 s before beginning of contraction. Certainly, this synchronization needs learning the correct technique. In spite of this learning, the exact performance is difficult for mothers practically [2, 5, 7, 10, 11]. In our primitive clinical assessment, we found out that some mothers were more eager to use continuous versus intermittent method. Similarly, Arthurs and Rosen proved that the mothers and midwives were more satisfied with continuous method [12]. It may have as a result of its easier usage, more painless effect and cause a nonstress status during application [13, 14].

As many midwives are afraid of maternal complications with continuous method, their repeated commemoration to mothers about special manner of using gas leads to fatigue and anxiety for not only mothers but also health staff. Since there are limited comparative surveys attributed to both methods of Entonox, we decided to compare these two methods in terms of obstetric outcomes to demonstrate whether the continuous method is as safe as intermittent method or not.

## 2. Patients and Methods

This randomized controlled clinical trial was conducted in Sabzevar Mobini Hospital in Iran in 2013. After taking the approval of the ethics committee and obtaining the informed consent of patients, a total of hundred women admitted for vaginal delivery were enrolled in the study. This number of sampling was determined by confidence coefficient of 95% and power of 80%. The inclusion criteria were singleton pregnancy, cephalic presentation, and term gestation. The exclusion criteria were macrosomia, maternal contracted pelvis, repeated cesarean section, unconfident FHR, spo_2_ less than 95%, and contraindications of Entonox usage including head injury, severe asthma, and also inability or unwillingness of patients to use inhalational gas.

The mothers were divided into two groups by simple randomization. One group including 50 women received Entonox intermittently and another group containing 50 women used it continuously. Before participating in the study, a comprehensive interview was conducted with mothers and registered in the checklist. Training of mothers was performed by a midwife (sampler). In intermittent group, mothers were breathing in mask during uterine contractions and put it aside between them. In continuous group, mothers were using gas constantly. The gas inhalation was started by commencement of active phase (cervical dilatation 3-4 cm and effacement 40–50%) and terminated by full dilatation of cervix. In all of the cases, maternal conditions influenced by Entonox were written in study forms. Also labor progression, fetal condition, and maternal O_2_ saturation were monitored and registered. The subjects concerning obstetric outcome including necessity to vacuum, perineal laceration, and uterine atony and newborn Apgar were recorded. Also maternal pushing collaboration was pointed in a prepared scale form by a midwife observing the delivery while she did not know which method of Entonox has been used for the parturient. The degree of satisfaction was scored by the mothers as well. Finally, statistical analysis was performed by spss 17 software, *t*-test, and Chi^2^ while *P* < 0.05 was considered significant.

## 3. Discussion and Results

The findings showed that demographic data and obstetric and fetal characteristics were matched in two groups (Table 1).

Spo_2_ was more than 95% in both groups and had no significant difference (*P* > 0.05). Adverse effects of Entonox were not significantly different in two groups (*P* > 0.05).

The mean duration of second stage of labor was 34 minutes in continuous group and 30 minutes in intermittent group; there is no significant difference (*P* = 0.3). The necessity of using oxytocin in second stage had no significant difference between two groups (*P* = 0.2).

4% of intermittent group needed assisted vaginal delivery (vacuum) in contrast to continuous group who delivered without any help; there is no significant difference (*P* = 0.4).

**Figure 1:**
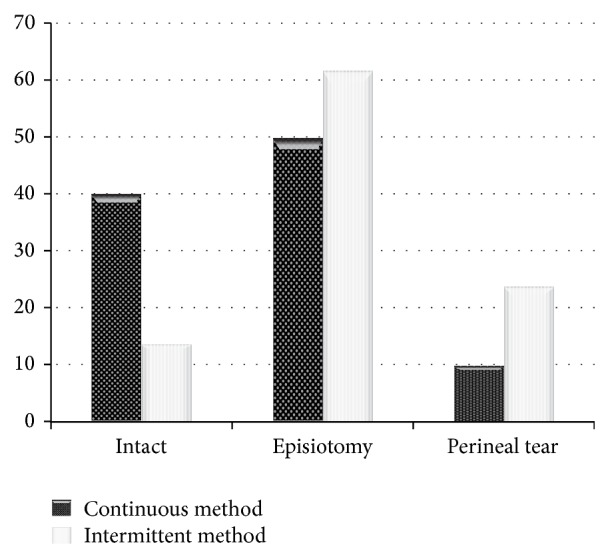
Comparison of perineal laceration rate in continuous versus intermittent method of Entonox.

This study showed that perineal lacerations were higher in intermittent group compared with continuous group significantly (*P* = 0.04) (Figure 1).

In 4% of intermittent group postpartum hemorrhage happened due to uterine atony in contrast to continuous group in which post-partum hemorrhage was 0%; there is no significant difference (*P* = 0.2).

Maternal collaboration in pushing during delivery which was pointed in the scale by the midwife was significantly more in continuous group (*P* = 0.03). Satisfaction scored by mothers was 96% in continuous group in contrast to 70% in intermittent group; there is significant difference (*P* < 0.0001).

**Figure 2:**
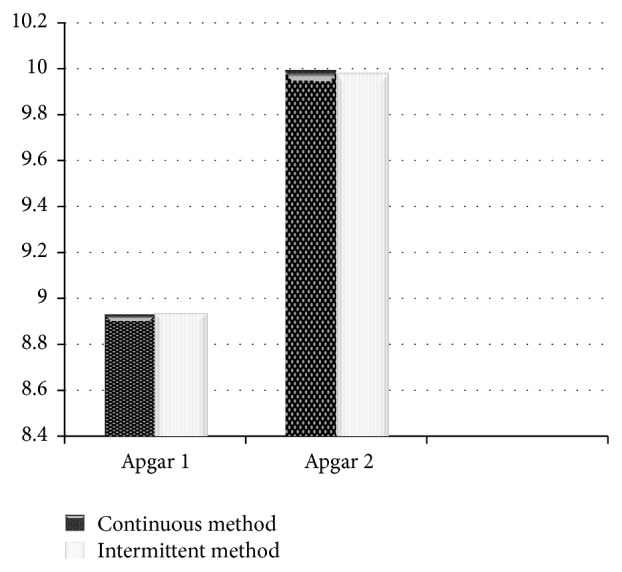
Comparison of Apgar in the first and fifth minutes between two groups.

Apgar score of neonates at first and fifth minute was acceptable and had no significant difference in two groups (*P* = 0.3) (Figure 2).

Efficacy and safety of Entonox for pain relief in labor have been proved for several decades. It is customary to use Entonox intermittently due to fear of maternal adverse effects by continuous method [13]. The aim of this study was to evaluate safety of continuous method in comparison with intermittent one. Previous researchers declared that Entonox is mostly used intermittently because continuous method leads to maternal drowsiness [8, 11]; however, our study showed that this complication was not so significant to avoid offering the continuous method to the mothers.

Our study showed that Entonox in each method does not have any adverse effects on delivery process. It did not increase duration of second stage of labor in both methods. Also, assisted vaginal birth was not increased in both methods; it was even accompanied with better results in continuous method. It is in agreement with other researchers that declared that using Entonox is safe even through second and third stages of labor [4]. According to previous reports, Entonox has no negative effect on breathing, circulation, and pushing or other bodily functions [3]. Our participants showed acceptable status during labor in both methods as well and even the continuous group had more collaboration in pushing by a significant difference.

Based on our findings, the continuous group faced less perineal lacerations than intermittent group. In our belief, higher maternal cooperation was effective in lower incidence of perineal lacerations. It is in agreement with Arthurs and Rosen and Zare Tazarjani et al. that declared continuous method is not harmful for mothers [12, 15].

In our study, uterine atony decreased by continuous method. However two methods were not associated with massive postpartum hemorrhage. Similarly, Arthurs and Rosen, Esfandiari et al., and Najafian et al. announced no severe maternal complications such as uterine atony by either continuous or intermittent method [12, 16, 17].

By a brief comparison with other analgesic methods, although Entonox in every manner does not omit pain completely, it has some advantages, which makes it worthy of usage. Although epidural in comparison with Entonox has more analgesic effect, it has some disadvantages including prolongation of labor, need for use of oxytocin, more assisted vaginal birth (vacuum or forceps), maternal hypotension, and urinary retention [4]. On the other hand, our study showed that Entonox does not impair pushing ability of mother during delivery and does not affect labor duration. Another benefit compared with epidural is that Entonox does not block motor nerves, so maternal ambulation is not limited, whereas inability to move with epidural analgesia leads to maternal fatigue and also retardation of fetal descent during labor. This subject plus decreased pushing force prolong labor duration and cause more need to using vacuum [3, 4]. In our investigation, none of the participants of continuous group of Entonox required vacuum.

*Pethidine* (another analgesic agent) can induce maternal confusion, sedation, respiratory depression, and hypoventilation that may confer with enough maternal collaboration during delivery [18], whereas our findings showed that Entonox had no adverse effect on maternal respiratory status, especially in continuous group that demonstrated more collaboration during delivery.

Finally, based on our experience, mothers' satisfaction rate was considerable with Entonox, worthy of attention that it was very prominent in continuous group (96%) in comparison with intermittent method (70%). This higher satisfaction rate was similar to results of Arthurs and Rosen that showed that continuous method was more desirable and 96% of mothers wished to continue offering this method, whereas 78% of mothers were satisfied with intermittent method [12]. By comparison, in the studies of Iravani and Salahian et al., respectively, satisfaction rate was 96% and 42% in intermittent group [19, 20]. The high satisfaction with continuous method may be due to some reasons, including more analgesic effect [13], easier usage, and less maternal anxiety [14, 21].

At last, in agreement with other researchers that declare that using Entonox is economical and affordable for all classes of the society [22, 23], our experience also revealed that the mothers of any educational and economical classes had good acceptance and cooperation with two methods especially in continuous group with a significant difference.

Totally, in our observation, continuous method is safe for both mother and baby. In comparison with intermittent method, it is accompanied with less maternal labor injury and results in more maternal compliance and satisfaction.

## 4. Conclusion

Entonox is a well-known inhalational agent for relieving labor pain. It is commonly used intermittently, which is associated with fatigue and anxiety for both mothers and health staff. Our results revealed acceptable obstetric outcomes by continuous method. By further investigations we would be able to let the mothers be free to choose the desired method of Entonox during labor.

## Figures and Tables

**Table 1:** Demographic characteristics of the participants.

Characteristics	Continuous	Intermittent	*P* value
Age (mother)	24.7 ± 4.0	24.8 ± 5.7	0.8
Age (husband)	28.9 ± 4.7	30.3 ± 5.9	0.2
Body mass index (kg/m^2^)	24.2 ± 2.9	24.8 ± 3.0	0.3
Educational level (mother)			
Primary (%)	60%	66%	0.5
Secondary (%)	14%	16%	
University (%)	26%	10%	
Educational level (husband)			
Primary (%)	48%	58%	0.7
Secondary (%)	52%	42%	
Annual household income			
Less than 200 dollars	72%	88%	0.3
More than 200 dollars	28%	12%	
Parity			
Primipara (%)	54%	58%	0.3
Multipara (%)	46%	42%	
Gestation (wk)	40.3 ± 1.2	40.9 ± 1.7	0.2
Birth weight	3245.4 ± 436.9	3097.4 ± 357.1	0.06
